# From where did the 2009 'swine-origin' influenza A virus (H1N1) emerge?

**DOI:** 10.1186/1743-422X-6-207

**Published:** 2009-11-24

**Authors:** Adrian J Gibbs, John S Armstrong, Jean C Downie

**Affiliations:** 1 Australian National University Emeritus Faculty, ACT, 0200, Australia; 2 CIDMLS, ICPMR, Westmead Hospital, NSW, 2145, Australia

## Abstract

The swine-origin influenza A (H1N1) virus that appeared in 2009 and was first found in human beings in Mexico, is a reassortant with at least three parents. Six of the genes are closest in sequence to those of H1N2 'triple-reassortant' influenza viruses isolated from pigs in North America around 1999-2000. Its other two genes are from different Eurasian 'avian-like' viruses of pigs; the NA gene is closest to H1N1 viruses isolated in Europe in 1991-1993, and the MP gene is closest to H3N2 viruses isolated in Asia in 1999-2000. The sequences of these genes do not directly reveal the immediate source of the virus as the closest were from isolates collected more than a decade before the human pandemic started. The three parents of the virus may have been assembled in one place by natural means, such as by migrating birds, however the consistent link with pig viruses suggests that human activity was involved. We discuss a published suggestion that unsampled pig herds, the intercontinental live pig trade, together with porous quarantine barriers, generated the reassortant. We contrast that suggestion with the possibility that laboratory errors involving the sharing of virus isolates and cultured cells, or perhaps vaccine production, may have been involved. Gene sequences from isolates that bridge the time and phylogenetic gap between the new virus and its parents will distinguish between these possibilities, and we suggest where they should be sought. It is important that the source of the new virus be found if we wish to avoid future pandemics rather than just trying to minimize the consequences after they have emerged. Influenza virus is a very significant zoonotic pathogen. Public confidence in influenza research, and the agribusinesses that are based on influenza's many hosts, has been eroded by several recent events involving the virus. Measures that might restore confidence include establishing a unified international administrative framework coordinating surveillance, research and commercial work with this virus, and maintaining a registry of all influenza isolates.

## Introduction

A novel H1N1 influenza virus, Swine-Origin Influenza Virus (S-OIV), was first isolated in mid-April 2009 and, by the end of the month, the first complete genomic sequences were published, and the virus shown to be of a novel re-assortant [1]. The virus spread fast in the human population, and the resulting pandemic has already proved to be a significant and very costly cause of mortality and morbidity in the human population. It has created intense interest worldwide. Several hundred research papers, reports, reviews and summaries [2,3] have been published about this virus in the last six months. Many discuss its genealogy deduced from its gene sequences, however it seems that we have no clearer evidence of its immediate origins than we have of the influenzas that caused past influenza pandemics. So the search for its source must be intensified while the clues are still fresh. The possibility that human activity may have had some role in its origins should not be dismissed without a dispassionate analysis of all available evidence. If we wish to avoid future pandemics, rather than just minimizing the damage they cause, we must better understand what conditions produce them.

Several phylogenetic studies of the gene sequences of S-OIV and other influenzas have now been reported [4-10]. In these studies the sequences have been compared using various techniques (e.g. statistical inference (SI), neighbour-joining, maximum parsimony and principal components analyses), and have involved various selections of the very large number of influenza gene sequences that are now publicly available. Most phylogenetic studies compared nucleotide sequences, and at least one compared the encoded amino acid sequences.

All studies have concluded that S-OIV emerged into the human population on a single occasion, probably around January 2009 [8,11]. They agree that six of its genes, those encoding the polymerase proteins (PB2, PB1 and PA), the haemagglutinin (HA), the nucleoprotein (NP) and the non-structural proteins (NS), show a clear affinity with those of the 'triple-reassortant' influenza viruses first found in North American pigs around 1998, whereas the other two genes, those encoding the neuraminidase (NA) and matrix proteins (MP), are from the Eurasian 'avian-like' virus lineage first isolated in Europe around 1979 [12-19]. Neither the 'triple- reassortant' viruses nor their individual genes have previously been found in Europe nor, likewise, have those of the 'avian-like' lineage been found in North America. However, viruses of both lineages have been found more recently in South East Asia [20,21], but reasortants intermediate between S-OIV and its parental lineages have not [22].

## Discussion

### Phylogenetic Studies

One of the most intriguing findings of the phylogenetic studies is that each S-OIV gene is connected to its respective phylogenetic tree by a noticeably long branch. This indicates that, immediately before its emergence, each had a period of "unsampled ancestry", which Smith and his colleagues estimated to be between 9.2 and 17.2 years long for the different genes [8]. Garten and her colleagues concluded however that "Though long, these branch lengths are not unusual for swine viruses; there are 52 other similar or longer branch lengths in the swine phylogenetic trees" that they published. However, Garten and her colleagues compared viruses collected over nine decades under a wide range of sampling intensities. It would be more appropriate to compare phylogenetically close isolates collected around the same time. We looked at branch lengths in a maximum likelihood tree of 160 HA nucleotide sequences most closely related to those of S-OIV, and found that after the long branch to the S-OIV HA gene cluster, the next longest branch was 79 isolates away and the next a further 21. There is a clear contrast between the branch lengths in trees of diverse sequences, and those in sister and cousin lineages.

**Figure 1:**
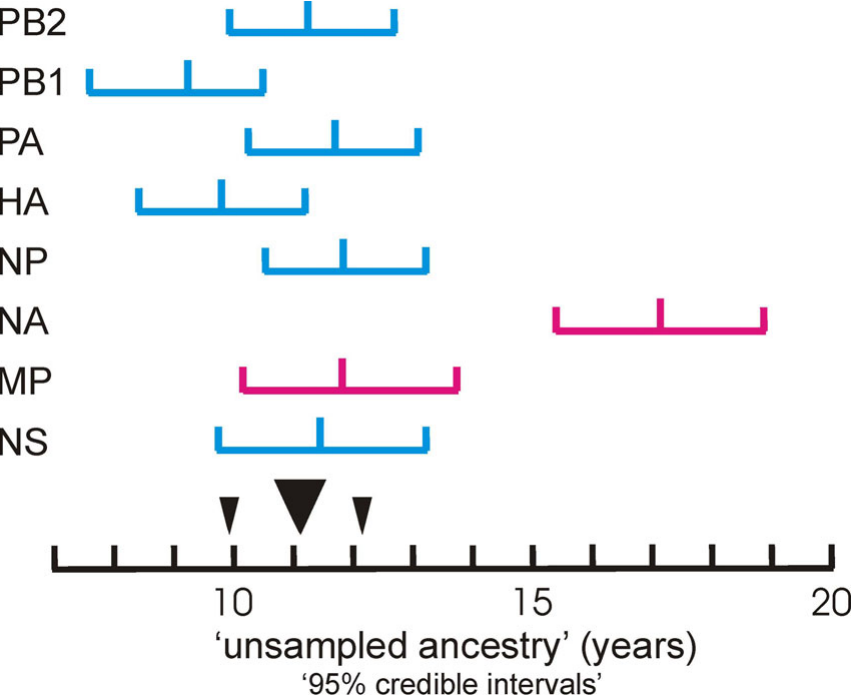
**Graph showing the duration of the "unsampled ancestry of different S-OIV genes**. The data is from Table 1 of [8], and the error bars give the "95% credible intervals". The data from the lineage of the triple reassortant parent are in blue, those from the Eurasian 'avian-like' swine virus lineage in red. The black triangles indicate the mean and standard deviation of the values for the triple reassortant and MP lineage genes combined.

Another unusual feature of the long S-OIV branches [8] is that the lengths and error ranges of the branches of seven of the genes estimated by Smith and colleagues (Table 1 in [8]) form a single broadly overlapping cluster (Fig. 1) with a mean length of 11.02 +/- 1.05 years, whereas those of the NA gene are significantly longer and indicate that it had not been sampled for more than 17.15 +/- 1.74 years. Thus although the NA and MP genes of S-OIV were both from the Eurasian avian-like lineage of influenza viruses, they probably first became associated with the S-OIV lineage on separate occasions, and hence came from two different viruses. We conclude therefore that S-OIV probably had at least three immediate parents, not two.

**Table 1:** Distances between genes of A/California/04/2009 (H1N1) and those of the closest H1N2 isolates.

		PB2^1^	PB1	PA	HA	NP	NS^3^
A/swine/Indiana/P12439/2000		0.0368*^2^	0.0430	0.0474	0.0525	0.0360*	0.0533

A/swine/Indiana/9K035/1999		0.0406	0.0395*	0.0498	0.0513*	0.0387	0.0439

A/swine/Minnesota/55551/2000		0.0372	0.0451	0.0450*	0.1224	0.0408	0.0427*

A/swine/Illinois/100084/2001		0.0446	0.0434	0.0451	0.0597	0.0404	0.0528

A/swine/Illinois/100085A/2001		0.0453	0.0420	0.0486	0.0578	0.0404	0.0539

^1 ^Genes: PB2, PB1 and PA, polymerase genes; HA, haemagglutinin; NP, nucleoprotein; NS, non-structural proteins 1 and 2

^2 ^Patristic distances; nucleotide substitutions/site. The isolate that gave the shortest distance for each gene is marked with an asterisk.

^3 ^All five NS1 genes encode a slightly truncated (219 amino acids) NS1 protein as does the NS1 gene of S-OIV.

The reports of the phylogenetic studies disagree most obviously in the sequences found to be closest to those of S-OIV. This is not surprising because the studies analysed different sets of sequences selected in different ways and different analytical techniques were used. Furthermore, the statistical inference methods used by some may not be ideal for identifying close neighbours in large datasets; evolution is stochastic, and close relationships are not statistical, and so a tree fitted statistically to a very large number of sequences [8] probably confounds close phylogenetic relationships, especially when those relationships have been globally optimized [7]. We therefore checked whether more consistent information about S-OIV's immediate parental lineages could be obtained from its gene sequences by using a more selective and direct approach. We first used SWeBLAST [23], a variant of BLAST, to select from the Genbank database only the sequences closest to those of S-OIV, then inferred their relationships using a maximum likelihood method, and finally ranked the sequences by their patristic distances within the trees using PATRISTIC [24].

**Figure 2:**
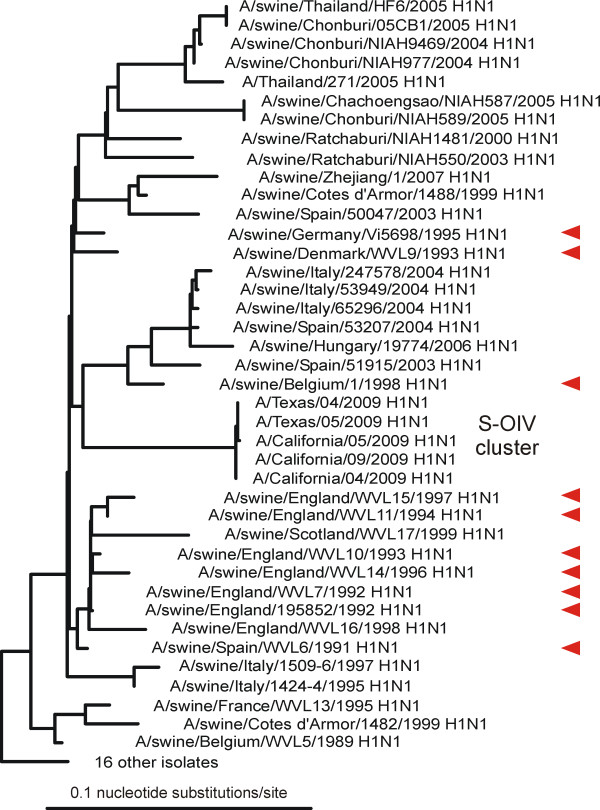
**Unrooted maximum likelihood tree of the neuraminidase gene sequences of S-OIV and the most closely related sequences in Genbank**. The ten closest are marked with red arrows. Details of the sequence selection and tree inference methods used are in Additional File 1.

**Figure 3:**
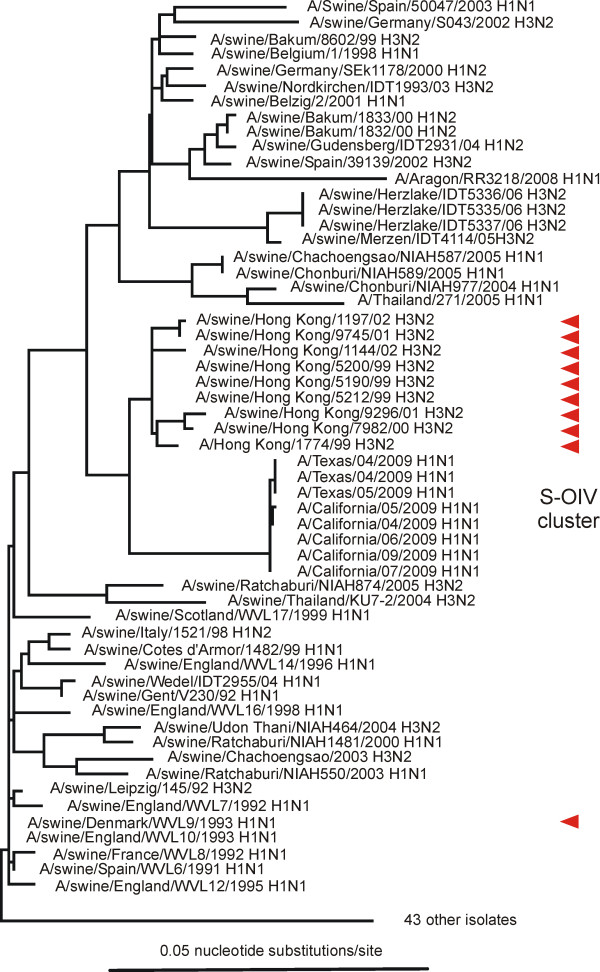
**Unrooted maximum likelihood tree of the gene sequences of the matrix proteins of S-OIV and the most closely related sequences in Genbank**. The ten closest are marked with red arrows. Details of the sequence selection and tree inference methods used are in Additional File 1.

**Figure 4:**
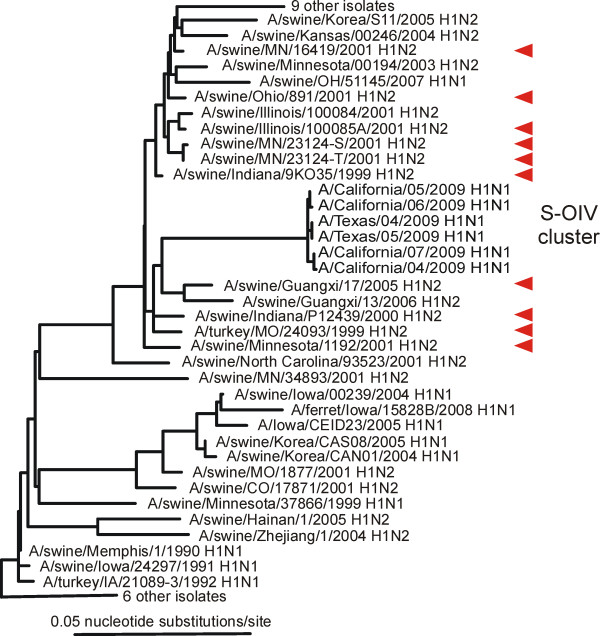
**Unrooted maximum likelihood tree of the haemagglutinin gene sequences of S-OIV and the most closely related sequences in Genbank**. The ten closest are marked with red arrows. Details of the sequence selection and tree inference methods used are in Additional File 1.

Our analyses showed that almost all the closest genes came from pig isolates. The NA genes closest to the 04/2009 NA were all from 'avian-like' H1N1 isolates from Europe sampled around 1991 (Figure 2) and Additional file 1; closest were A/swine/Spain/WVL6/1991 (H1N1) (0.0706 nucleotide substitutions/site: ns/s), A/swine/England/WVL7/1992 (H1N1) (0.0718ns/s) and A/swine/England/WVL10/1993 (H1N1) (0.0753ns/s). The MP genes closest to the 04/2009 MP gene were also from 'avian-like' isolates but collected in Asia around 1999 (Figure 3); closest were A/swine/Hong Kong/5200/1999 (H3N2), A/swine/Hong Kong/51901999 (H3N2) and A/swine/Hong Kong/5212/1999 (H3N2) (all 0.0254 ns/s). The other six genes, including the HA gene (Figure 4), were closest to those of North American 'triple-reassortant' isolates sampled around or soon after 1999; most were H1N2 isolates from pigs, although a few of the polymerase genes were close to H3N2 isolates (data not shown). We narrowed the search for the triple reassortant parent by assuming, as is likely, that the six S-OIV genes came from a single triple reassortant rather than two or more. We found that there were five triple-reassortant isolates with four or five genes that were among the twenty closest to those of 04/2009 (Table 1). Closest of all were A/swine/Indiana/9K035/1999 (H1N2), A/swine/Indiana/P12439/2000 (H1N2) and A/swine/Minnesota/55551/2000 (H1N2).

So, in summary, our analyses provide consistent evidence that the immediate parents were swine viruses. The sampling dates of those isolates are congruent with the estimated lengths of 'unsampled ancestry' of the parents [8] and, together with differences in provenance support the conclusion that S-OIV had three parents; one from North America, one from Europe and the third from Asia.

## Hypotheses

The results of the phylogenetic analyses outlined above can be used to construct plausible scenarios of the ways in which S-OIV might have originated. This is a useful exercise as it may focus the search for new clues. Some of the crucial evidence provided by the phylogenetic analyses is that:

1) S-OIV emerged into the human population on a single occasion, probably in Mexico.

2) S-OIV is a reassortant with at least three parental viruses, all of them viruses of pigs.

3) the parents of S-OIV were last sampled directly in three very distant parts of the world.

4) the parental genes were last sampled more than a decade ago. Two were sampled around 11 years ago, the third 17 years ago, whereas their sister and cousin lineages have been sampled frequently.

S-OIV could have been generated by natural means. The parental isolates could, for example, have been assembled in one place by migratory birds, however the consistent link of S-OIV's immediate ancestors with pigs suggests that human activity of some sort was involved in bringing together the parental viruses. At least two contrasting theories are congruent with this possibility and the available clues:

1) The *"*unsampled pig herd" theory was suggested by Smith and his colleagues [8], who concluded that "the progenitor of the S-OIV epidemic originated in pigs", and the "long unsampled history observed for every segment" of the S-OIV genome "suggests that the reassortment of Eurasian and North American swine lineages may not have occurred recently, and it is possible that this single reassortant lineage has been cryptically circulating rather than two distinct lineages of swine flu", and that "Movement of live pigs between Eurasia and North America seems to have facilitated the mixing of diverse swine influenzas, leading to the multiple reassortment events associated with the genesis of the S-OIV strain."

This theory was implicitly supported by Trifonov and his colleagues in their report of a study of the numbers of gene sequences deposited in Genbank from human and pig influenzas sampled around the world in different years [25]. They had found that, although the number of influenza sequences deposited had increased greatly in the last decade, there were four times as many human as pig influenza sequences. Furthermore, whereas the sequenced human isolates came from all over the world, the pig isolates came only from North America, Asia, and Europe, and none from Africa, Oceania, or South America. They concluded that, given "the lack of sampling" of pigs "in certain parts of the world, it is perhaps not surprising that the ancestors of the new human influenza A (H1N1) virus have gone unnoticed for almost two decades." [26].

It is important to note that this theory depends on the intercontinental movement of live infected pigs, and requires at least two quarantine-breaching incursions involving three different countries. It is likely that quarantine control of the spread of swine influenzas around the world varies greatly in its efficacy. However viruses of the Eurasian 'avian-like' lineage, and their genes, have never been found in North America before S-OIV appeared, even though they have been common in Europe for over three decades, and similarly 'triple reassortant' viruses and their genes have not been isolated in Europe, although they have been the dominant swine influenza virus in North America for more than a decade.

2) The "laboratory error" theory. We note that influenza viruses survive well in virus laboratories, that laboratories are not subject to routine surveillance, and that there are probably many laboratories in the world that share and propagate a range of swine influenza viruses from different sources and continents, and also share and use immortalized lines of cultured cells. The viruses are used for research, diagnostic tests and for making vaccines, and the cells are used for propagating the viruses. Thus if S-OIV had been generated by laboratory activity, when one host was simultaneously infected with strains from the different parental lineages, this would explain most simply why S-OIV's genes had escaped surveillance for over a decade, and how viruses last sampled in North America, Europe and Asia became assembled in one place and generated a reassortant.

So what sort of laboratory event might produce mixed infections with different strains of influenza, and thereby generate S-OIV? The simplest is that S-OIV is a reassortant produced during research. There is also the possibility that it was generated during the production of multivalent vaccines. Multivalent 'killed' vaccines are mixtures of virions that have been grown in hen's eggs and then chemically sterilized. Thus a reassortant might be produced if insufficient sterilant, usually formaldehyde or propiolactone, is added to the virion mixture. The live mixture could then infect pigs 'vaccinated' with it, and the growing viruses could reassort, infect piggery staff and hence spread to the broader human population. Finally, it is possible that serially passaged cells, such as the Madin-Darby canine kidney (MDCK) cells now widely used in influenza laboratories, became latently and serially infected with different strains of influenza as a result of lax laboratory practices. This process could generate reassortants, and infect staff.

## Circumstantial Evidence

There are clear historical precedents for most of the events described in the above scenarios. Viruses do 'escape' from laboratories, even high security facilities. The H1N1 influenza lineage that circulated in the human population for four decades after the 1918 Spanish influenza epidemic, disappeared during the 1957 Asian influenza pandemic, was absent for two decades, but then reappeared in 1977. Gene sequences of the 1977 isolate and others collected in the 1950s were almost identical, indicating that the virus had not replicated and evolved in the interim, and had probably been held in a laboratory freezer between 1950 and 1977 and 'escaped' during passaging. The suggestion that persistently infected cells might be involved is also not outlandish; influenza virus can persistently and latently infect MDCK cells [27], and viruses do travel between laboratories in cells [28].

Multivalent 'killed' vaccines are widely used to control swine influenzas, particularly in North American piggeries [29], indeed one of the viruses identified by us and others (e.g. [30]) as closest to S-OIV, A/swine/Indiana/P12439/2000 (H1N2), seems to be the "2000 Indiana strain" used in commercial vaccines in North America [31]. We also note that isolates selected from the three clusters of viruses we find to be closest to S-OIV would probably make a useful trivalent vaccine for international use as they would provide a mixture of haemagglutinins of the swine H3, H1 'classical swine' and H1 'Eurasian avian-like' lineages.

The patchy occurrence of S-OIV infections in piggeries over the past six months is interesting and may be significant. Pigs have been shown to be fully susceptible to S-OIV. They shed the virus and readily transmit it between themselves, but whereas S-OIV has been reported in humans worldwide, it has not yet been reported from a pig farm in the USA (October 2009). By contrast it has been found in two piggeries each in Australia, Canada and Ireland, and one each in Argentina, Indonesia and Japan. In the outbreaks in Argentina, Australia and Canada, at least, the pigs had not been vaccinated (Jorge H. Dillon, J. Keenliside and Alain Laperle, personal communication), and became infected from infected farm staff. The apparent immunity to S-OIV of pigs in the USA and Mexico, but not elsewhere, may indicate that the swine influenza vaccines currently used in the USA and Mexico contain an immunogen that either protects against S-OIV infection or mitigates its symptoms.

Circumstantial evidence must always be treated with caution. One major uncertainty in trying to determine the origin of S-OIV is that one cannot predict which characters of the parental viruses have remained or changed during the reassortment process that produced S-OIV. If, for example, the significant infectiousness of S-OIV is an 'emergent' property of S-OIV, and not shown by its parents, then one could conclude that the final reassortment probably occurred at about the time it emerged in early 2009. However it is not yet known whether S-OIV's infectiousness is novel; the reassortment may have occurred a decade ago, and a recent mutation may have enhanced its infectiousness. Another widely reported feature of S-OIV is that it replicates poorly in embryonated eggs, but again this may be merely a specific feature of S-OIV and not its immediate parents. Similarly the fact that the evolutionary rate of all of the genes of S-OIV seem to be 'normal' during their unsampled pre-emergent period [8,11]] does not prove that the virus or its parents have been maintained in "unsampled" pig herds and precluded the possibility of human involvement, as viruses grown for vaccines evolve, and indeed might be expected to show an increased evolutionary rate [32,33] while adapting to eggs, a new host, although such an increase may have been offset by the practice of storing 'seed stocks' for use in several 'production cycles' in vaccine production, so that the evolutionary age of a vaccine virus may be less than its sidereal age, and the average could then appear to be 'normal'. Finally there is the report that the first human S-OIV infections were in Perote, a small Mexican town with a very large number of large piggeries, although it was also reported that none of the pigs showed signs of influenza. Among the earliest cases were some in Oaxaca, 290 kms to the south [34]. Perote is an unlikely place for an infected migratory pig to arrive from an intercontinental trip, as the town is in a remote high valley surrounded by mountains, 200 kms to the east of Mexico City where there is the nearest major airport, and 130 kms from the nearest port at Vera Cruz. The four month difference between 'The Most Recent Common Ancestor' date for S-OIV estimated from its phylogeny [8,11], and its earliest detection in the human population makes it more difficult to make specific conclusions about its provenance.

## Motifs and Sequence Signatures

We have also checked whether any extra information about the origin of S-OIV can be gleaned from gene sequence features reported to be associated with host adaptation, virulence, etc. Such sequence signatures must be interpreted with caution as although Genbank records the source host of influenza isolates, it rarely records their passage hosts and passage history. Influenza viruses are nowadays mostly isolated in MDCK cells, but early influenza isolates were mostly grown in embryonated hen's eggs, and adaptation to eggs is known to cause protein sequence changes [32,33,35,36]. Therefore we compared sequence signatures and motifs in S-OIV with those of their closest relatives.

Subbarao and his colleagues [37] were first to show that amino acid 627 of the PB2 protein was almost always glutamate in bird isolates and lysine in human isolates. We checked 142 PB2 sequences, about one third each of isolates from birds, pigs and human beings, and found glutamate in contrast to lysine at this site in 98% and 70% of the bird and pig isolates. The only human isolates that had glutamate were all five S-OIVs, and three other human isolates; A/Hong Kong/156/1997(H5N1) and A/Hong Kong/1073/1999(H9N2) both from human beings infected from birds, and A/Hong Kong/1774/1999(H3N2) which came from a person infected from pigs. Chen and colleagues [38] made a much more extensive survey of sequences and found 51 more sites in 10 of the 11 proteins of influenza virus that discriminated between bird and human isolates as well as, or better than, PB2-627. Unfortunately they did not report similarly specific sites for swine isolates, but we have checked whether any of those 52 sites (Table 1 in [38]) also distinguish S-OIV and its closest relatives, and found that only two of the 52 sites, PA-356 and NP-313, did. At 29 of the sites, the amino acids of the 'S-OIV cluster' (i.e. S-OIV and the swine viruses closest to it) are avian-like, at 16 they are human-like, at 6 (in the matrix proteins) they are novel, and the single recognised site in some NS1s has been lost by truncation. However, surprisingly, all the five recognised sites in the PB1-F2 protein of the S-OIV cluster have human-like residues, whereas the other 11 human-like residues are spread over 40 sites in eight proteins.

Another oddity of the S-OIV genome is that its PB1-F2 gene is truncated. In most influenza viruses the PB1 gene encodes three proteins [39,40]. The primary ORF encodes the PB1 and PB1-N40 proteins, and the PB1-F2 ORF, which encodes a proapoptotic protein of 90 amino acids, is in the second (+1) reading frame of the gene starting at nt 95. In a small number of influenzas, including all S-OIVs, the PB1-F2 ORF is truncated by termination codons at positions 12, 58 and 88, and its absence is associated with avirulence in mice [41-43]. Trifonov and colleagues have reported statistical tests of various features of the PB1-F2 region [26], and concluded "that PB1-F2 is of little or no evolutionary significance for the virus".

We compared the PB1-F2 genes of S-OIV with those most closely related to them. Four of the five triple-reassortants closest to S-OIV (Table 1) have a complete PB1-F2, but one, A/swine/Minnesota/55551/2000, terminates at codon 58 and so is partly truncated. The PB1-F2 of another isolate, A/swine/Minnesota/3236/2007 (H1N2) has termination codons 12, 26 and 58 and, together with A/swine/Ohio/75004/2004 (H1N1), which has termination codon 58, forms a distant sister group to the S-OIVs in a ML tree of the complete PB1 genes. A survey of the individual S-OIV PB1-F2 termination codons in 7644 PB1-F2 sequences (Genbank; August 2009) established that one might expect to find, at random, 0.46 sequences with all three termination codons in a dataset of that size, whereas they were found not only in S-OIV but also in the unrelated A/mallard/Alberta/300/1977 (H1N1) and A/Siena/9/1989 (H1N1). The termination codons in the S-OIV PB1-F2 originate as silent mutations to valine or leucine (VL) codons of the main PB1 ORF, but whereas VL codons are evenly distributed throughout the PB1 protein, the termination mutations in the +1 frame are not; three of the eleven VL codons have mutated in the PB1-F2 region, which is 90 codons long, but only in two of the 82 VL codons in the remaining 665 codons have mutated. It seems that the peculiarities of the S-OIV PB1-F2 gene, the human-like signature sites and its selectively super-imposed termination codons, probably reflect the outcome of selection rather than being of "little or no evolutionary significance".

Finally, we examined the NS1 protein, which in c. 80% of over 3000 sequences obtained from Genbank (July 2009) were full length, and at the C-terminus had an intact '-ESEV' motif or a similar sequence, which has been linked with virulence [44]. 7% of the NS1s were, like that of S-OIV, only 219 amino acids long and terminating in '-QK'; most of them (66%) had come from pig isolates, 20% from human, 8% birds, 5% horses and fewer than 1% from mink and dogs, but none were as short as the NS1 protein experimentally truncated to 126 amino acids to attenuate the virus for use in a live vaccine [45].

Thus our examination of sequence signatures and motifs in the S-OIV genome has not clarified our knowledge of its origins, but has certainly raised many new questions.

## Conclusion

Influenza virus is a very significant zoonotic pathogen. Public confidence in influenza research, and the agribusinesses that are based on influenza's many hosts, has been eroded by several recent events. Measures that might restore confidence include establishing both a unified international administrative framework coordinating all surveillance, research and commercial work with this virus, and also a detailed registry of all influenza isolates held for research and vaccine production.

The phylogenetic information presently available does not identify the source of S-OIV, however it provides some clues, which can be translated into hypotheses of where and how it might have originated. Two contrasting possibilities have been described and discussed in this commentary, but more data are needed to distinguish between them. It would be especially valuable to have gene sequences of isolates filling the time and phylogenetic gap between those of S-OIV and those closest to it. We believe that these important sequences are most likely to be found in isolates from as-yet-unsampled pig populations or as-yet-unsampled laboratories, especially those holding isolates of all three clusters of viruses closest to those of S-OIV, and involved in vaccine research and production. Quarantine and trade records of live pigs entering North America could probably focus the search for the unsampled pig population. It is likely that further information about S-OIV's immediate ancestry will be obtained when the unusual features of its PB1-F2 gene are understood.

## Abbreviations

BLAST: Basic Local Alignment Search Tool; ORF: open reading frame.

## Competing interests

The work reported here was unfunded, and the authors have no competing financial or intellectual property interests.

## Authors' contributions

The work was planned as a result of discussions and projects involving all the authors. Analyses were done by AJG, all authors contributed to the manuscript.

## Supplementary Material

Additional file 1 **Taxonomic methods and sequence Accession Codes**. Details of the methods used to produce Figs 2, 3 and 4 together with a listing of the Accession Codes of all the sequences used in the analyses. Click here for file

## References

[B1] Dawood FS Jain S Finelli L Shaw MW Lindstrom S Garten RJ Gubareva LV Xu X Bridges CB Uyeki TM Emergence of a novel swine-origin influenza A (H1N1) virus in humans New England Journal of Medicine 2009 361 10.1056/NEJMoa0903810 19423869

[B2] Cohen J Straight from the pig's mouth: swine research with swine influenzas Science 2009 325 140 141 10.1126/science.325_140 19589977

[B3] Hensley SE Yewdell JW Que sera, sera: evolution of the swine H1N1 influenza A virus Expert Review of Anti-infective Therapy 2009 7 763 768 10.1586/eri.09.62 19735217 PMC2943383

[B4] Chen J-M Sun Y-X Chen J-W Liu S Yu J-M Shen C-J Sun X-D Peng D Panorama phylogenetic diversity and distribution of type A influenza viruses based on their six internal gene sequences Virology Journal 2009 6 137 10.1186/1743-422X-6-137 19737421 PMC2746212

[B5] Liu S Ji K Chen J Tai D Jiang W Hou G Chen J Li J Huang B Panorama phylogenetic diversity and distribution of type A influenza Virus PLoS ONE 2009 4 e5022 10.1371/journal.pone.0005022 19325912 PMC2658884

[B6] Babakir-Mina M Dimonte S Perno CF Ciotti M Origin of the 2009 Mexico influenza virus: a comparative phylogenetic analysis of the principal external antigens and matrix protein Archives of Virology 19582546 10.1007/s00705-009-0438-1

[B7] Garten RJ Davis CT Russell CA Shu B Lindstrom S Balish A Sessions WM Xu X Skepner E Deyde V Antigenic and genetic characteristics of swine-origin 2009 A(H1N1) influenza viruses circulating in humans Science 2009 325 197 201 10.1126/science.1176225 19465683 PMC3250984

[B8] Smith GJD Vijaykrishna D Bahl J Lycett SJ Worobey M Pybus OG Ma SK Cheung CL Raghwani J Bhatt S Origins and evolutionary genomics of the 2009 swine-origin H1N1 influenza A epidemic Nature 2009 10.1038/nature08182 19516283

[B9] Nava GM Attene-Ramos MS Ang JK Escorcia M Origins of the new influenza A (H1N1) virus: time to take action Eurosurveillance 2009 14 1 22 10.2807/ese.14.22.19228-en 19497253

[B10] Solovyov A Palacios G Briese T Lipkin WI Rabadan R Cluster analysis of the origins of the new influenza A(H1N1) virus Eurosurveillance 2009 14 1 55 10.2807/ese.14.21.19224-en PMC4310691 19480812

[B11] Fraser C Donnelly CA Cauchemez S Hanage WP Van Kerkhove MD Hollingsworth DT Griffin J Baggaley RF Jenkins HE Lyons EJ Pandemic potential of a strain of influenza A (H1N1): early findings ScienceExpress 2009 10.1126/science.1176062 PMC3735127 19433588

[B12] Brockwell-Staats C Webster RG Webby RJ Diversity of influenza viruses in swine and the emergence of a novel human pandemic influenza A (H1N1) Influenza and Other Respiratory Viruses 2009 3 207 213 10.1111/j.1750-2659.2009.00096.x 19768134 PMC2746644

[B13] Shinde V Bridges CB Uyeki TM Shu B Balish A Xu X Lindstrom S Gubareva LV Deyde V Garten RJ Triple-reassortant swine influenza A (H1) in humans in the United States, 2005-2009 New England Journal of Medicine 2009 361 10.1056/NEJMoa0903812 19423871

[B14] Olsen CW The emergence of novel swine influenza viruses in North America Virus Research 2002 85 199 210 10.1016/S0168-1702(02)00027-8 12034486

[B15] Richt JA Lager KM Janke BH Woods RD Webster RG Webby RJ Pathogenic and antigenic properties of phylogenetically distinct reassortant H3N2 swine influenza viruses cocirculating in the United States Journal of Clinical Microbiology 2003 41 3198 3205 10.1128/JCM.41.7.3198-3205.2003 12843064 PMC165376

[B16] Pensaert M Ottis K Vanderputte J Kaplan MM Buchmann PA Evidence for the natural transmission of influenza A virus from wild ducks to swine and its potential for man Bulletin of the World Health Organisation 1981 59 75 78 PMC2396022 6973418

[B17] Brown IH Ludwig S Olsen CW Hannoun C Scholtissek C Hinshaw VS Harris PA McCauley JW Strong I Alexander DJ Antigenic and genetic analyses of H1N1 influenza A viruses from European pigs Journal of General Virology 1997 78 553 562 9049404 10.1099/0022-1317-78-3-553

[B18] Scholtissek C Burger H Bachmann PA Hannoun C Genetic relatedness of hemagglutinins of the H1 subtype of influenza A viruses isolated from swine and birds Virology 1983 129 521 523 10.1016/0042-6822(83)90194-0 6623931

[B19] Webster RG Bean WJ Gorman OT Chambers TM Kawaoka Y Evolution and ecology of influenza A viruses Microbiological Reviews 1992 56 152 179 1579108 10.1128/mr.56.1.152-179.1992 PMC372859

[B20] Pascua PNQ Songa M-S Lee JH Choi H-W Han JH Kim J-H Yoo G-J Kim C-J Choi Y-K Seroprevalence and genetic evolutions of swine influenza viruses under vaccination pressure in Korean swine herds Virus Research 2008 138 43 49 10.1016/j.virusres.2008.08.005 18789984

[B21] Yu H Hua R-H Zhang Q Liu T-Q Liu H-L Li G-X Tong G-Z Genetic evolution of swine influenza A (H3N2) viruses in China from 1970 to 2006 Journal of Clinical Microbiology 2008 46 1067 1075 10.1128/JCM.01257-07 18199784 PMC2268354

[B22] Kingsford C Nagarajan N Salzberg SL 2009 Swine-Origin Influenza A (H1N1) resembles previous influenza isolates PLoS ONE 2009 4 e6402 10.1371/journal.pone.0006402 19636415 PMC2712239

[B23] Fourment M Gibbs AJ Gibbs MJ SWeBLAST: A Sliding Window Web-based BLAST Tool for recombinant analysis Journal of Virological Methods 2008 152 98 101 10.1016/j.jviromet.2008.06.009 18602951

[B24] Fourment M Gibbs MJ PATRISTIC: a program for calculating patristic distances and graphically comparing the components of genetic change BMC Evolutionary Biology 2006 6 1 10.1186/1471-2148-6-1 16388682 PMC1352388

[B25] Trifonov V Khiabanian H Rabadan R Geographic pependence, surveillance, and origins of the 2009 influenza A (H1N1) virus New England Journal of Medicine 2009 361 115 119 10.1056/NEJMp0904572 19474418

[B26] Trifonov V Racaniello V Rabadan R The contribution of the PB1-F2 protein to the fitness of influenza A viruses and its recent evolution in the 2009 influenza A (H1N1) pandemic virus PLoS Currents: Influenza 2009 22 10.1371/currents.RRN1006 PMC2762337 20029605

[B27] Tobita K Tanaka T Hayase Y Spontaneous excretion of virus from MDCK cells persistently infected with influenza virus A/PR/8/34 Journal of General Virology 1997 78 563 566 9049405 10.1099/0022-1317-78-3-563

[B28] Stang A Petrasch-Parwez E Brandt S Dermietzel R Meyer HE Stühler K Liffers S-T Überla K Grunwald T Unintended spread of a biosafety level 2 recombinant retrovirus Retrovirology 2009 6 68 10.1186/1742-4690-6-86 19772602 PMC2760500

[B29] Mackenzie D Swine flu: the predictable pandemic? New Scientist 2009 2706 6 7

[B30] Gallaher WR Towards a sane and rational approach to management of influenza H1H1 2009 Virology Journal 2009 6 51 10.1186/1743-422X-6-51 19422701 PMC2691743

[B31] Anonymous American Association of Veterinary Virologists 2009 http://www.aasp.org/public/H1N1InfluenzaVeterinaryTalkingPoints-05-05-09.doc

[B32] Bush RM Fitch WM Bender CA Cox NJ Positive selection on the H3 hemagglutinin gene of human influenza virus A Molecular Biology and Evolution 1999 16 1457 1465 10555276 10.1093/oxfordjournals.molbev.a026057

[B33] Bush RM Smith CB Cox NJ Fitch WM Effects of passage history and sampling bias on phylogenetic reconstruction of human influenza A evolution Proceedings of the National Academy of Sciences, USA 2000 97 6974 6980 10.1073/pnas.97.13.6974 PMC34372 10860959

[B34] Cohen J Interview with head of Mexico's top swine flu lab ScienceInsider 2009 http://blogs.sciencemag.org/scienceinsider/2009/05/exclusive-inter.html

[B35] Robertson JS Clinical influenza virus and the embryonated hen's egg Reviews in Medical Virology 1993 3 97 106 10.1002/rmv.1980030206

[B36] Widjaja L Ilyushina N Webster RG Webby RJ Molecular changes associated with adaptation of human influenza A virus in embryonated chicken eggs Virology 2006 350 137 145 10.1016/j.virol.2006.02.020 16545416

[B37] Subbarao EK London W Murphy BR A single amino acid in the PB2 gene of influenza A virus is a determinant of host range Journal of Virology 1993 67 1761 1764 8445709 10.1128/jvi.67.4.1761-1764.1993 PMC240216

[B38] Chen GW Chang SC Mok CK Lo YL Kung YN Huang JH Shih YH Wang JY Chiang C Chen CJ Shih SR Genomic signatures of human versus avian influenza A viruses Emerging Infectious Diseases 2006 12 1353 1360 17073083 10.3201/eid1209.060276 PMC3294750

[B39] Wise HM Foeglein A Sun J Dalton RM Patel S Howard W Anderson EC Barclay WS Digard P A complicated message: identification of a novel PB1-related protein translated from influenza A virus segment 2 mRNA Journal of Virology 2009 83 8021 8031 10.1128/JVI.00826-09 19494001 PMC2715786

[B40] McAuley JL Hornung F Boyd KL Smith AM McKeon R Bennink J Yewdell JW McCullers JA Expression of the 1918 influenza A virus PB1-F2 enhances the pathogenesis of viral and secondary bacterial pneumonia Cell Host and Microbe 2007 2 240 249 10.1016/j.chom.2007.09.001 18005742 PMC2083255

[B41] Conenello GM Palese P Influenza A virus PB1-F2: a small protein with a big punch Cell Host and Microbe 2007 2 207 209 10.1016/j.chom.2007.09.010 18005736

[B42] Conenello GM Zamarin D Perrone LA Tumpey T Palese P A single mutation in the PB1-F2 of H5N1 (HK/97) and 1918 influenza A viruses contributes to increased virulence PLoS Pathogens 2007 3 e141 10.1371/journal.ppat.0030141 PMC2000966 17922571

[B43] Zamarin D Ortigoza MB Palese P Influenza A virus PB1-F2 protein contributes to viral pathogenesis in mice Journal of Virology 2006 80 7976 7983 10.1128/JVI.00415-06 16873254 PMC1563817

[B44] Krug RM Yuan W Noah DL AG L Intracellular warfare between human influenza viruses and human cells: the roles of the viral NS1 protein Virology 2003 309 181 189 10.1016/S0042-6822(03)00119-3 12758165

[B45] Richt JA Lekcharoensuk P Lager KM Vincent AL Loiacono CM Janke BH Wu W-H Yoon K-J Webby RJ Solórzano A García-Sastre A Vaccination of pigs against swine influenza viruses by using an NS1-truncated modified live-virus vaccine Journal of Virology 2006 80 11009 11018 10.1128/JVI.00787-06 16943300 PMC1642165

