# Supplementary material for: From where did the 2009 'swine-origin' influenza A virus (H1N1) emerge?
Source: Virol J. 2009 Nov 24;6:207. doi: 10.1186/1743-422X-6-207 (PMC2787513; doi:10.1186/1743-422X-6-207)
Supplement: Additional file 1 — Taxonomic methods and sequence Accession Codes. Details of the methods used to produce Figs 2, 3 and 4 together with a listing of the Accession Codes of all the sequences used in the analyses. [file 1743-422X-6-207-S1.DOC]

The Genbank nucleotide database was searched using SWeBLAST [1] on 29 April 2009 for sequences matching each of the eight genes of the first fully sequenced isolate of the Swine-Origin Influenza Virus (S-OIV), A/California/04/2009. The SWeBLAST program sends short overlapping subsequences of the query sequence (100 nts window, step 25 nts) to the BLAST facility of Genbank, and collates and ranks the results. This system provides a simple method for detecting recombinant sequences, but none were found. It also identifies, for each query subsequence, all the most closely related sequences. The closest five non-S-OIV sequences for each SWeBLAST subsequence, together with all the S-OIV sequences, then available, were collated, replicates removed, aligned using MAFFT [2] and edited to obtain the coding regions.

This search strategy ensured that all the closest sequences in the database are found, but limits the number of more distant sequences, as these confound close relationships. The SWeBLAST search for sequences close to the NA and MP sequences of A/California/04/2009 was repeated on 14^th^ July 2009, and the data for the MP sequences combined.

The phylogenetic relationships of sequences were inferred and compared using the maximum likelihood method PhyML [3] with the general time-reversible model with gamma-distributed rate variation with a proportion of invariable sites (GTR+I+G). and all other optional parameters estimated by the program. The resulting trees were viewed using TREEVIEW, were converted to patristic distances using PATRISTIC [4] and sorted using Microsoft EXCEL. As a check, trees were also calculated using the neighbor-joining method [5], and trees with closely similar topologies and clusterings were obtained. The resulting datasets of the most closely related sequences contained from 52 to 131 sequences for each gene:

|  | Accession Code^1^ | Number of S-OIV seqs | Number of non S-OIV seqs |
| --- | --- | --- | --- |
| PB2^2^ | FJ966079 | 4 | 108 |
| PB1 | FJ966080 | 4 | 127 |
| PA | FJ966081 | 5 | 115 |
| HA | FJ966082 | 6 | 46 |
| NP | FJ966083 | 5 | 66 |
| NA | FJ966084 | 5 | 51 |
| MP | FJ966085 | 7 | 57 |
| NS | FJ966086 | 2 | 55 |

^1^ The Accession codes of all sequences in the datasets are given below.

^2^ PB2, PB1 and PA are the polymerase genes; HA, the haemagglutinin; NP, the nucleoprotein; NA, the neuraminidase; MP, the matrix 1 and 2; NS, the non-structural 1 and 2 genes.

PB2 sequences:

AF098587, AF098588, AF251410, AF251418, AF251426, AF251434, AF455731, AF455732, AF455733, AF455734, AF455735, AF455736, AF455737, AF455738, AF457697, AY129163, AY233387, AY703829, CY004427, CY005173, CY005177, CY013870, CY014813, CY015491, CY016147, CY016195, CY016618, CY017780, CY017868, CY018892, CY020268, CY020956, CY020988, CY021628, CY021700, CY021708, CY022164, CY032878, CY032879, CY032880, CY033629, CY033794, CY035414, CY035417, CY035420, CY035423, CY035427, CY035430, CY035433, CY035436, CY035439, CY035443, CY035445, CY035486, CY038233, CY039358, DQ150422, DQ150430, DQ280213, DQ469963, DQ923520, EF551050, EU015993, EU026089, EU084946, EU182250, EU258942, EU301177, EU399758, EU409946, EU409953, EU604691, EU743217, EU743313, EU743535, EU743559, EU798919, EU798920, EU798922, EU798925, EU798926, EU798927, EU798928, EU798929, EU798930, EU798931, EU798932, EU798933, EU798934, EU798935, EU798936, EU798937, EU871843, EU871875, EU980486, FJ374512, FJ461607, FJ517281, FJ517305, FJ517309, FJ517341, FJ610101, FJ686753, FJ686761, FJ686769, FJ686777, FJ686785, FJ686793, FJ966079, FJ966955, FJ966963, FJ966976

PB1 sequences:

AF037420, AF037421, AF250130, AF342823, AF455726, AF455727, AF455728, AF455729, AF455730, AY129162, AY233388, CY001283, CY001419, CY001830, CY002222, CY003614, CY003630, CY006377, CY006553, CY006809, CY008954, CY008970, CY008986, CY009490, CY009746, CY009994, CY010010, CY010066, CY010522, CY010730, CY011462, CY011470, CY011822, CY011894, CY012222, CY012230, CY012238, CY012478, CY012502, CY012566, CY012574, CY012686, CY012902, CY012918, CY012974, CY013333, CY013483, CY013917, CY016074, CY016489, CY016513, CY016521, CY016553, CY016585, CY017297, CY017305, CY033612, CY033790, CY035028, CY035140, CY035204, CY035228, CY035236, CY035415, CY035424, CY035428, CY035431, CY035434, CY035440, CY035446, CY036837, CY036853, CY036909, CY036989, CY037493, CY038509, CY038605, CY038757, CY038917, CY038941, CY038965, CY038973, CY038981, CY038997, CY039005, CY039013, CY039061, CY039085, CY039093, CY039117, CY039165, CY039181, DQ145544, DQ280206, DQ280214, DQ469972, DQ889683, DQ923518, DQ923519, EF551051, EF597436, EU015992, EU258941, EU301400, EU409954, EU409959, EU604692, EU798898, EU798899, EU798900, EU798901, EU798903, EU798904, EU798905, EU798906, EU798907, EU798908, EU798909, EU798910, EU798911, EU798912, EU798913, EU798914, EU798915, EU798916, EU798917, FJ374513, FJ966080, FJ966958, FJ966965, FJ966978

PA sequences:

AF455717, AF455718, AF455720, AF455722, CY004618, CY004930, CY005055, CY005374, CY012805, CY014707, CY015448, CY017778, CY017858, CY018890, CY018906, CY021258, CY021474, CY029846, CY029934, CY035378, CY035418, CY035421, CY035425, CY035429, CY035432, CY035435, CY035438, CY035441, CY035444, CY035447, CY035707, CY035710, CY035876, CY036579, CY036595, CY036611, CY036658, CY036756, CY036764, CY036788, CY036796, CY037108, CY038235, CY038245, CY038361, CY038369, CY038377, CY038385, CY039332, CY039364, DQ145539, DQ150432, DQ889684, DQ923516, DQ923517, EF492344, EF551052, EF597413, EF634339, EU015991, EU084929, EU182252, EU301368, EU557453, EU557459, EU557467, EU557471, EU557472, EU604693, EU735823, EU735831, EU735839, EU743024, EU743103, EU743111, EU743215, EU743278, EU798878, EU798879, EU798880, EU798881, EU798882, EU798883, EU798884, EU798885, EU798886, EU798887, EU798888, EU798889, EU798890, EU798891, EU798892, EU798893, EU798894, EU798895, EU798896, EU798897, EU871889, EU980471, FJ374517, FJ432775, FJ432783, FJ461603, FJ517264, FJ517319, FJ517328, FJ610085, FJ686703, FJ686711, FJ686719, FJ686735, FJ686799, FJ686807, FJ686815, FJ966081, FJ966957, FJ966964, FJ966970, FJ966977, M26085

HA sequences:

AF250124, AF455675, AF455677, AF455680, AF455681, AF455682, AY038014, AY060046, AY060047, AY060048, AY060049, AY060050, AY060051, AY060052, CY027155, CY027507, CY035070, DQ139320, DQ280195, DQ280203, DQ666933, DQ889689, EF556199, EF556201, EF556203, EU139827, EU139828, EU139830, EU139831, EU139832, EU604689, EU735786, EU743159, EU798778, EU798779, EU798780, EU798781, EU798782, EU798783, EU798784, EU798785, EU798786, EU798787, EU798788, FJ357104, FJ750522, FJ966082, FJ966952, FJ966959, FJ966960, FJ966974, FJ966982

NP sequences :

AB434377, AB434409, AF250127, AF251407, AF251415, AF251423, AF251431, AF455702, AF455704, AF455705, AF455706, AY129159, CY014763, CY022432, CY022472, CY022480, CY022965, CY024928, CY025013, CY027158, CY028783, CY028791, CY035073, CY036802, DQ145541, DQ280193, DQ280217, DQ469991, DQ469999, EF551054, EU015990, EU258936, EU258946, EU301304, EU604694, EU697205, EU697210, EU735821, EU735829, EU743154, EU743162, EU743178, EU743213, EU798844, EU798849, EU798850, EU798851, EU798852, EU798853, EU798854, EU798855, EU798856, EU798857, EU850621, EU850624, EU871831, FJ357107, FJ374515, FJ461599, FJ789828, FJ789833, FJ966083, FJ966953, FJ966961, FJ966967, FJ966979, L11164, L46849, M63755, M76602, M76610

NA sequences:

AB292403, AB294216, AB434290, AB434298, AB434306, AB434314, AB434322, AB434330, AF250366, AJ410876, AJ410880, AJ410881, AJ410883, AJ412689, AJ412690, AJ412692, AJ416626, AM920729, CY009894, CY010574, CY010582, CY022988, CY025255, CY037900, CY037908, CY037916, CY037931, CY037938, CY037946, CY037954, CY037962, CY037969, CY037977, CY037985, CY037993, CY038001, CY038009, CY038017, CY038025, EF101756, EF124312, EF124325, EF541474, EU045388, EU045389, EU045393, EU296600, EU296602, EU296604, FJ415611, FJ688267, FJ798780, FJ805963, FJ966084, FJ966956, FJ966969, FJ966973, FJ966981

MP sequences:

AJ293925, AJ316047, AJ316052, AJ316059, AM157383, AM157384, AM746618, AY363573, AY363574, AY363575, AY363576, AY363577, AY363578, AY363579, AY363580, AY664425, CY009381, CY009629, CY009893, CY014740, CY014748, CY015040, CY015082, CY015121, CY020822, CY020830, CY020838, CY020854, CY022646, CY026428, CY027292, CY030996, CY031004, CY031141, CY032214, DQ186976, DQ186977, DQ186978, DQ186981, DQ186982, DQ186983, DQ186984, DQ997456, EF101742, EF101750, EU152199, EU516314, EU636687, EU636695, EU742621, EU743355, EU743387, EU743403, EU871922, EU980524, FJ966085, FJ966954, FJ966962, FJ966968, FJ966972, FJ966975, FJ966980, FJ966983, Z46439

NS sequences :

AB434348, AF153263, AF250128, AF455710, AF455711, AY060129, AY060130, AY060131, AY060132, AY060133, AY060134, AY060138, AY060141, AY060144, AY060145, AY060146, AY060147, AY060148, AY060149, AY129160, AY619978, AY619979, CY022433, CY028784, CY035074, DQ280240, DQ280255, EF455566, EU015988, EU399755, EU604696, EU697208, EU697213, EU735790, EU735822, EU743155, EU743163, EU743214, EU798858, EU798859, EU798865, EU798866, EU798867, EU798868, EU798870, EU798871, EU798872, EU798875, EU798877, EU826547, EU826555, FJ357108, FJ374516, FJ789836, FJ789837, FJ966086, FJ966966

**References**

1. Fourment M, Gibbs AJ, Gibbs MJ: **SWeBLAST: A Sliding Window Web-based BLAST Tool for recombinant analysis.** *Journal of Virological Methods* 2008, **152:**98-101.

2. Katoh K, Asimenos G, Toh H: **Multiple Alignment of DNA Sequences with MAFFT.** In *Methods in Molecular Biology.* *Volume* 537. Edited by Posada D; 2009: 39-64

3. Guindon S, Gascuel O: **PhyML - A simple, fast, and accurate algorithm to estimate large phylogenies by maximum likelihood.** *Systematic Biology* 2003, **52:**696-704.

4. Fourment M, Gibbs MJ: **PATRISTIC: a program for calculating patristic distances and graphically comparing the components of genetic change.** *BMC Evolutionary Biology* 2006, **6:**1.

5. Saitou N, Nei M: **The neighbor-joining method: a new method for reconstructing phylogenetic trees.** *Molecular Biology and Evolution* 1987, **4:**406-425.
